# 3,3′-Ethyl­enebis(3,4-dihydro-6-chloro-2*H*-1,3-benzoxazine)

**DOI:** 10.1107/S1600536810014248

**Published:** 2010-04-21

**Authors:** Augusto Rivera, Jicli José Rojas, Jaime Ríos-Motta, Michal Dušek, Karla Fejfarová

**Affiliations:** a Departamento de Química, Universidad Nacional de Colombia, Bogotá, AA 14490, Colombia; b Institute of Physics, Na Slovance 2, 182 21 Praha 8, Czech Republic

## Abstract

The asymmetric unit of the title compound, C_18_H_18_Cl_2_N_2_O_2_, contains one half of an independent mol­ecule, the other half being generated *via* a centre of inversion at the mol­ecular centroid. In the crystal structure, mol­ecular chains are formed through non-classical C—H⋯ O hydrogen bonds between an axial H atom of the oxazine ring and the O atom of a neighbouring mol­ecule.

## Related literature

For the synthesis, see: Rivera *et al.* (1989 ▶). For related structures, see: Rivera *et al.* (1986 ▶); Huerta *et al.* (2006 ▶); Chen & Wu (2007 ▶); Ranjith *et al.* (2009 ▶). For uses of benzoxazines in polymer science, see Yaggi *et al.* (2009 ▶). For the biological activity of bis-benzoxazine compounds, see: Billmann & Dorman (1963 ▶); Heinisch *et al.* (2002 ▶).
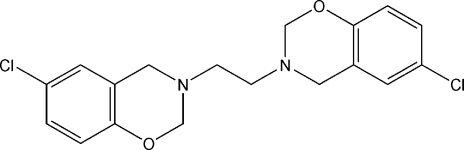


## Experimental

### 

#### Crystal data

C_18_H_18_Cl_2_N_2_O_2_
*M* *_r_* = 365.3
Monoclinic, 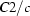

*a* = 18.9920 (5) Å
*b* = 5.8884 (2) Å
*c* = 17.8813 (5) Å
β = 125.449 (4)°
*V* = 1629.03 (12) Å^3^
*Z* = 4
Cu *K*α radiation
μ = 3.70 mm^−1^
*T* = 120 K
0.30 × 0.19 × 0.12 mm

#### Data collection

Oxford Diffraction Xcalibur diffractometer with an Atlas (Gemini ultra Cu) detector
Absorption correction: analytical [*CrysAlis PRO* (Oxford Diffraction, 2009 ▶), using a multifaceted crystal model based on expressions derived by Clark & Reid (1995 ▶)] *T*_min_ = 0.593, *T*_max_ = 0.787
12716 measured reflections
1442 independent reflections
1344 reflections with *I* > 3σ(*I*)
*R*_int_ = 0.024

#### Refinement

*R*[*F* ^2^ > 2σ(*F* ^2^)] = 0.030
*wR*(*F* ^2^) = 0.103
*S* = 2.26
1442 reflections
109 parameters
H-atom parameters constrained
Δρ_max_ = 0.25 e Å^−3^
Δρ_min_ = −0.25 e Å^−3^

### 

Data collection: *CrysAlis CCD* (Oxford Diffraction, 2009 ▶); cell refinement: *CrysAlis RED* (Oxford Diffraction, 2009 ▶); data reduction: *CrysAlis RED*; program(s) used to solve structure: *SIR2002* (Burla *et al.*, 2003 ▶); program(s) used to refine structure: *JANA2006* (Petříček *et al.*, 2006 ▶); molecular graphics: *DIAMOND* (Brandenburg & Putz, 2005 ▶); software used to prepare material for publication: *JANA2006*.

## Supplementary Material

Crystal structure: contains datablocks global, I. DOI: 10.1107/S1600536810014248/fj2292sup1.cif

Structure factors: contains datablocks I. DOI: 10.1107/S1600536810014248/fj2292Isup2.hkl

Additional supplementary materials:  crystallographic information; 3D view; checkCIF report

## Figures and Tables

**Table 1:** Hydrogen-bond geometry (Å, °)

*D*—H⋯*A*	*D*—H	H⋯*A*	*D*⋯*A*	*D*—H⋯*A*
C2—H2B⋯O1^i^	0.96	2.56	3.369 (2)	142

Symmetry code: (i) 
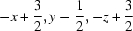
.

**Fig. 1.**
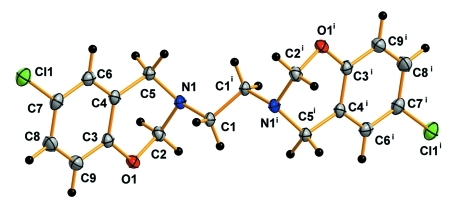
The molecular structure of title compound, showing the atomic numbering scheme with atomic displacement ellipsoids drawn at the 50%.

**Fig. 2.**
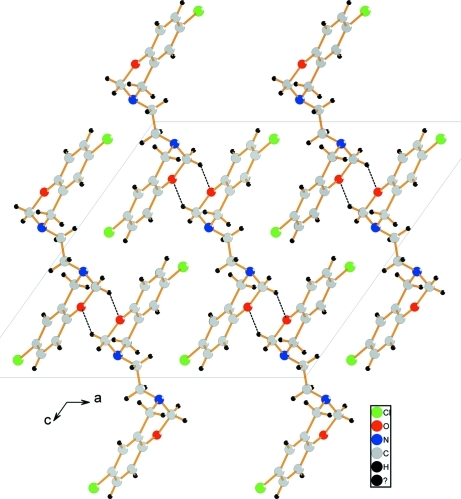
Perspective views of the crystal packing showing hydrogen-bonded interactions (dashed lines).

## supplementary crystallographic information

### Comment

1,3-Benzoxazines are heterocyclic compound obtained from condensation between phenols, formaldehyde and a primary amine. Applications of these compounds are in polymeric and pharmacological fields. Recently the structure of these compounds has attracted much attention see Huerta *et al.* (2006); Chen & Wu (2007); Ranjith *et al.* (2009). During our investigations, a series of bis-benzoxazines were prepared by reaction of phenols, formaldehyde and ethylenediamine (Rivera *et al.*, 1986). However, the crystallization of these compounds was difficult and led to crystals of bad quality. In the present work, the single crystals of the title compound were finally successfully prepared and its crystal structure has been determined herein.

The molecule contains two 1,3-benzoxazine units linked by an ethylene bridge. The asymmetric unit of the title compound C_18_H_18_Cl_2_N_2_O_2_, contains one-half of the formula unit; a centre of inversion is located at the mid-point of the central C1—C1^i^ bond (see Fig. 1). Both oxazine rings are in cyclohexene-like conformations with normal bond distances and angles, and their values were found in good agreement with the corresponding values in the related structures reported by Huerta *et al.* (2006), Chen & Wu (2007) and Ranjith *et al.* (2009). In the crystal structure, molecules are linked *via* C2—H2B···O1 weak hydrogen bonds forming a molecular slab (see Fig 2a,b). The bond involves axial-hydrogen of oxazine ring and the oxygen atom of a neighbor molecule.

There is also possibility for very weak intermolecular interaction between the hydrogen H2A and the aromatic ring C3,C4,C6, C7, C8, C9, with the distance between H2A and the centre of the ring of 2.99 Å.

### Experimental

Under vigorous stirring a mixture of ethylenediamine (0.34 ml, 5 mmol) and *p*-chlorophenol (1.3 g 10 mmol) was dissolved in dioxane (10 ml) and (1.5 ml, 20 mmol) was slowly added. Stirring was continued for 4 h at rt until a precipitate appeared. The solid was filtered off and washed with water (1.83 g, 92%). Recrystallization from ethanol gave a white solid.

### Refinement

All hydrogen atoms were discernible in difference Fourier maps and could be refined to reasonable geometry. According to common practice H atoms attached to C atoms were nevertheless kept in ideal positions during the refinement. The isotropic atomic displacement parameters of hydrogen atoms were evaluated as 1.2**U*_eq_ of the parent atom.

### Crystal data

**Table:**

C_18_H_18_Cl_2_N_2_O_2_	*F*(000) = 760
*M**_r_* = 365.3	*D*_x_ = 1.489 Mg m^−^^3^
Monoclinic, *C*2/*c*	Cu *K*α radiation, λ = 1.54184 Å
Hall symbol: -C 2yc	Cell parameters from 10117 reflections
*a* = 18.9920 (5) Å	θ = 3.0–66.8°
*b* = 5.8884 (2) Å	µ = 3.70 mm^−^^1^
*c* = 17.8813 (5) Å	*T* = 120 K
β = 125.449 (4)°	Prism, colorless
*V* = 1629.03 (12) Å^3^	0.30 × 0.19 × 0.12 mm
*Z* = 4	

### Data collection

**Table:**

Oxford Diffraction Xcalibur diffractometer with an Atlas (Gemini ultra Cu) detector	1442 independent reflections
Radiation source: X-ray tube	1344 reflections with *I* > 3σ(*I*)
mirror	*R*_int_ = 0.024
Detector resolution: 10.3784 pixels mm^-1^	θ_max_ = 75.1°, θ_min_ = 5.4°
Rotation method data acquisition using ω scans	*h* = −22→22
Absorption correction: analytical [*CrysAlis PRO* (Oxford Diffraction, 2009), using a multifaceted crystal model based on expressions derived by Clark & Reid (1995)]	*k* = −7→6
*T*_min_ = 0.593, *T*_max_ = 0.787	*l* = −19→20
12716 measured reflections	

### Refinement

**Table:**

Refinement on *F*^2^	36 constraints
*R*[*F*^2^ > 2σ(*F*^2^)] = 0.030	H-atom parameters constrained
*wR*(*F*^2^) = 0.103	Weighting scheme based on measured s.u.'s *w* = 1/[σ^2^(*I*) + 0.0016*I*^2^]
*S* = 2.26	(Δ/σ)_max_ = 0.014
1442 reflections	Δρ_max_ = 0.25 e Å^−^^3^
109 parameters	Δρ_min_ = −0.25 e Å^−^^3^
0 restraints	

### Special details

**Table:**

Experimental. CrysAlisPro (Oxford Diffraction Ltd., Version 1.171.33.51 (release 27-10-2009 CrysAlis171 .NET) Analytical numeric absorption correction using a multifaceted crystal model based on expressions derived by Clark & Reid (1995)
Refinement. The refinement was carried out against all reflections. The conventional *R*-factor is always based on *F*. The goodness of fit as well as the weighted *R*-factor are based on *F* and *F*^2^ for refinement carried out on *F* and *F*^2^, respectively. The threshold expression is used only for calculating *R*-factors etc. and it is not relevant to the choice of reflections for refinement.The program used for refinement, Jana2006, uses the weighting scheme based on the experimental expectations, see _refine_ls_weighting_details, that does not force *S* to be one. Therefore the values of *S* are usually larger than the ones from the SHELX program.

### Fractional atomic coordinates and isotropic or equivalent isotropic displacement parameters (Å^2^)

**Table:**

	*x*	*y*	*z*	*U*_iso_*/*U*_eq_	
Cl1	0.60192 (2)	−0.00670 (6)	0.93208 (3)	0.0280 (3)	
O1	0.67792 (6)	0.65142 (16)	0.72490 (7)	0.0215 (5)	
N1	0.60904 (7)	0.4270 (2)	0.58616 (8)	0.0180 (5)	
C1	0.53100 (8)	0.5511 (3)	0.54598 (10)	0.0185 (7)	
C2	0.67854 (8)	0.5625 (3)	0.64492 (10)	0.0198 (6)	
C3	0.65998 (9)	0.4905 (2)	0.77097 (11)	0.0192 (7)	
C4	0.62512 (8)	0.2799 (2)	0.73128 (10)	0.0190 (6)	
C5	0.61011 (8)	0.2223 (2)	0.63683 (10)	0.0191 (6)	
C6	0.60690 (8)	0.1294 (2)	0.78148 (10)	0.0208 (6)	
C7	0.62347 (9)	0.1873 (3)	0.86889 (10)	0.0233 (7)	
C8	0.65769 (9)	0.3956 (3)	0.90753 (11)	0.0261 (7)	
C9	0.67601 (10)	0.5459 (3)	0.85788 (11)	0.0246 (7)	
H1a	0.54087	0.707097	0.539047	0.0222*	
H1b	0.509368	0.533303	0.582633	0.0222*	
H2a	0.679796	0.688363	0.61157	0.0238*	
H2b	0.730991	0.479648	0.668433	0.0238*	
H5a	0.65391	0.118579	0.647102	0.023*	
H5b	0.556851	0.140341	0.598522	0.023*	
H6	0.582529	−0.016899	0.755597	0.0249*	
H8	0.668857	0.438584	0.965284	0.0313*	
H9	0.700365	0.691858	0.884131	0.0295*	

### Atomic displacement parameters (Å^2^)

**Table:**

	*U*^11^	*U*^22^	*U*^33^	*U*^12^	*U*^13^	*U*^23^
Cl1	0.0306 (3)	0.0293 (3)	0.0288 (3)	0.00199 (13)	0.0199 (2)	0.00560 (14)
O1	0.0192 (5)	0.0205 (6)	0.0179 (5)	−0.0041 (4)	0.0068 (4)	−0.0012 (4)
N1	0.0124 (5)	0.0177 (6)	0.0169 (6)	0.0007 (4)	0.0044 (5)	0.0005 (5)
C1	0.0141 (6)	0.0178 (7)	0.0161 (8)	0.0023 (5)	0.0045 (6)	0.0013 (6)
C2	0.0150 (6)	0.0227 (7)	0.0174 (7)	−0.0018 (5)	0.0069 (6)	−0.0001 (6)
C3	0.0137 (6)	0.0193 (8)	0.0183 (8)	0.0015 (4)	0.0057 (6)	0.0018 (5)
C4	0.0131 (6)	0.0203 (7)	0.0172 (7)	0.0028 (5)	0.0052 (5)	0.0006 (5)
C5	0.0162 (6)	0.0173 (7)	0.0176 (7)	0.0010 (5)	0.0062 (5)	0.0011 (5)
C6	0.0146 (6)	0.0193 (7)	0.0231 (8)	0.0016 (5)	0.0079 (6)	0.0007 (6)
C7	0.0194 (6)	0.0251 (8)	0.0248 (8)	0.0042 (5)	0.0125 (6)	0.0053 (6)
C8	0.0267 (7)	0.0282 (8)	0.0210 (8)	0.0026 (6)	0.0125 (6)	−0.0014 (6)
C9	0.0233 (7)	0.0217 (7)	0.0228 (8)	−0.0005 (6)	0.0100 (6)	−0.0023 (6)

### Geometric parameters (Å, °)

**Table:**

Cl1—C7	1.813 (2)	C3—C9	1.437 (3)
O1—C2	1.529 (2)	C4—C5	1.578 (3)
O1—C3	1.421 (2)	C4—C6	1.439 (3)
N1—C1	1.4182 (18)	C5—H5a	0.96
N1—C2	1.3690 (16)	C5—H5b	0.96
N1—C5	1.501 (2)	C6—C7	1.445 (3)
C1—C1^i^	1.4853 (18)	C6—H6	0.96
C1—H1a	0.96	C7—C8	1.372 (2)
C1—H1b	0.96	C8—C9	1.432 (3)
C2—H2a	0.96	C8—H8	0.96
C2—H2b	0.96	C9—H9	0.96
C3—C4	1.3907 (19)		
			
C2—O1—C3	116.65 (11)	C3—C4—C6	116.51 (16)
C1—N1—C2	110.35 (12)	C5—C4—C6	125.13 (12)
C1—N1—C5	111.32 (14)	N1—C5—C4	113.84 (12)
C2—N1—C5	109.60 (10)	N1—C5—H5a	109.4703
N1—C1—C1^i^	106.07 (13)	N1—C5—H5b	109.4713
N1—C1—H1a	109.4706	C4—C5—H5a	109.4721
N1—C1—H1b	109.4717	C4—C5—H5b	109.4707
C1^i^—C1—H1a	109.47	H5a—C5—H5b	104.7188
C1^i^—C1—H1b	109.4723	C4—C6—C7	123.28 (13)
H1a—C1—H1b	112.6688	C4—C6—H6	118.3591
O1—C2—N1	112.97 (15)	C7—C6—H6	118.3615
O1—C2—H2a	109.4708	Cl1—C7—C6	122.62 (11)
O1—C2—H2b	109.4711	Cl1—C7—C8	117.49 (15)
N1—C2—H2a	109.471	C6—C7—C8	119.88 (18)
N1—C2—H2b	109.4715	C7—C8—C9	117.04 (18)
H2a—C2—H2b	105.7297	C7—C8—H8	121.4818
O1—C3—C4	120.14 (17)	C9—C8—H8	121.4814
O1—C3—C9	120.27 (12)	C3—C9—C8	123.70 (14)
C4—C3—C9	119.58 (17)	C3—C9—H9	118.1485
C3—C4—C5	118.35 (16)	C8—C9—H9	118.1475
			
C2—N1—C1—C1^i^	150.21 (14)	C9—C3—C4—C6	0.2 (3)
C5—N1—C1—C1^i^	−87.87 (15)	O1—C3—C9—C8	178.38 (17)
C3—O1—C2—N1	46.41 (18)	C4—C3—C9—C8	−0.3 (3)
C2—O1—C3—C4	−14.6 (2)	C3—C4—C5—N1	−18.4 (2)
C2—O1—C3—C9	166.66 (16)	C6—C4—C5—N1	162.77 (15)
C1—N1—C2—O1	61.58 (16)	C3—C4—C6—C7	−0.3 (3)
C5—N1—C2—O1	−61.37 (17)	C5—C4—C6—C7	178.57 (16)
C1—N1—C5—C4	−74.43 (16)	C4—C6—C7—Cl1	−178.83 (13)
C2—N1—C5—C4	47.92 (19)	C4—C6—C7—C8	0.5 (3)
N1—C1—C1^i^—N1^i^	180.00 (13)	Cl1—C7—C8—C9	178.84 (14)
O1—C3—C4—C5	2.6 (2)	C6—C7—C8—C9	−0.5 (3)
O1—C3—C4—C6	−178.46 (15)	C7—C8—C9—C3	0.5 (3)
C9—C3—C4—C5	−178.74 (16)		

Symmetry codes: (i) −*x*+1, −*y*+1, −*z*+1.

### Hydrogen-bond geometry (Å, °)

**Table:**

*D*—H···*A*	*D*—H	H···*A*	*D*···*A*	*D*—H···*A*
C2—H2B···O1^ii^	0.96	2.56	3.369 (2)	142

Symmetry codes: (ii) −*x*+3/2, *y*−1/2, −*z*+3/2.

## References

[bb1] Billmann, J. H. & Dorman, L. C. (1963). *J. Med. Chem.* **6**, 701–708. 10.1021/jm00342a016 14184929

[bb2] Brandenburg, K. & Putz, H. (2005). *DIAMOND* Crystal Impact GbR, Bonn, Germany.

[bb3] Burla, M. C., Camalli, M., Carrozzini, B., Cascarano, G. L., Giacovazzo, C., Polidori, G. & Spagna, R. (2003). *J. Appl. Cryst.* **36**, 1103.

[bb4] Chen, X.-L. & Wu, M.-H. (2007). *Acta Cryst.* E**63**, o3684.

[bb5] Clark, R. C. & Reid, J. S. (1995). *Acta Cryst.* A**51**, 887–897.

[bb6] Heinisch, L., Wittmann, S., Stoiber, T., Berg, A., Ankel-Fuchs, D. & Mollmann, U. (2002). *J. Med. Chem.* **45**, 3032–3039. 10.1021/jm010546b 12086488

[bb7] Huerta, R., Toscano, R. A. & Castillo, I. (2006). *Acta Cryst.* E**62**, o2938–o2940.

[bb8] Oxford Diffraction (2009). *CrysAlis CCD*, *CrysAlis RED* and *CrysAlis PRO* Oxford Diffraction Ltd, Yarnto, England.

[bb9] Petříček, V., Dušek, M. & Palatinus, L. (2006). *JANA2006* Institute of Physics, Praha, Czech Republic.

[bb10] Ranjith, S., Thenmozhi, S., Manikannan, R., Muthusubramanian, S. & Subbiahpandi, A. (2009). *Acta Cryst.* E**65**, o581. 10.1107/S1600536809005790 PMC2968457 21582236

[bb11] Rivera, A., Aguilar, Z., Clavijo, D. & Joseph-Nathan, P. (1989). *Anal. Quim.* **85**, 9–10.

[bb12] Rivera, A., Ospina, E., Sanchez, A. & Joseph-Nathan, P. (1986). *Heterocycles*, **24**, 2507–2510.

[bb13] Yaggi, Y., Kiskan, B. & Ghosh, N. N. (2009). *J. Polym. Sci. Part A Polym. Chem.* **47**, 5565–5576.

